# Retraction: Dynamics of natural product Lupenone as a potential fusion inhibitor against the spike complex of novel Semliki Forest Virus

**DOI:** 10.1371/journal.pone.0336280

**Published:** 2025-11-10

**Authors:** The PLOS One Editors

After this article [1] was published, concerns were raised about Fig 4. Specifically, the three replicate simulations in each of Figs 4A-D appear similar.

During editorial follow-up, the corresponding authors NM and AA provided the individual-level quantitative data and raw log files underlying Fig 4, and additional information regarding the methodology used in the molecular dynamics simulations. They stated that similarity between replicates of each molecular dynamic simulation in Figs 4A-D was a possible result of using identical simulation parameters and use of the same computational model and software. PLOS consulted an Editorial Board member from *PLOS One*, who advised that the level of similarity observed between the replicate MD simulations suggests that the study design and methodology underlying Fig 4 are flawed. Due to unresolved concerns regarding the reliability of the triplicate simulation approach, the claims of reproducibility and the conclusions drawn from the MD studies are not supported.

PLOS additionally notes there are concerns about competing interests and peer review.

In light of the above unresolved concerns, the *PLOS One* Editors retract this article. We regret that the issues were not addressed prior to the article’s publication.

NM, ADas, SM, AG, AA, and DB did not agree with the retraction. PK, ADey, FA, PS, and MKAM either did not respond directly or could not be reached.
